# Influence of Endogenous Bacteria on Behavioral Responses in *Leptocybe invasa*: An Analysis of mVOCs

**DOI:** 10.3390/insects15060455

**Published:** 2024-06-16

**Authors:** Leming Zhou, Ping Hu, Jinting Xie, Junjue Li, Chunhui Guo, Zhengde Yang

**Affiliations:** 1 Guangxi Colleges and Universities Key Laboratory for Cultivation and Utilization of Subtropical Forest Plantation, Guangxi Key Laboratory of Forest Ecology and Conservation, College of Forestry, Guangxi University, Nanning 530004, China; zlm160826@outlook.com (L.Z.); huping@gxu.edu.cn (P.H.); xiejt333@163.com (J.X.); 13978755183@163.com (J.L.); 2 Ecological Environment Monitoring and Scientific Research Center, Yellow River Basin Ecology and Environment Administration, Ministry of Ecology and Environment, Zhengzhou 450004, China

**Keywords:** *Leptocybe invasa*, endogenous bacteria, GC–MS, insect behavior, Y-tube olfactometer

## Abstract

**Simple Summary:**

This study investigates the impact of the mVOCs emanating from nine cultivable endogenous bacteria within *Leptocybe invasa* on the host’s behavioral selection.

**Abstract:**

Microorganisms within insects play a vital role in maintaining the basal physiological functions of the insects, with olfactory signals as critical components of insect survival strategies. *Leptocybe invasa* (*L. invasa*), an invasive alien pest inflicting significant damage to eucalyptus trees, harbors a rich and varied bacterial community within its body. However, the impact of its endogenous bacteria and their microbial Volatile Organic Compounds (mVOCs) on the behavioral preferences of *L. invasa* remains unexplored to date. This study focused on nine cultivable and dominant endogenous bacterial strains within *L. invasa*. Using a Y-tube olfactometer, we investigated the behavioral responses of female *L. invasa* to the mVOCs emitted by these bacteria. Concurrently, gas chromatography–mass spectrometry (GC–MS) was employed to quantify the mVOCs produced by these endogenous bacteria. Our findings revealed that *Staphylococcus* sp. exhibited the highest attractiveness of *L. invasa*, whereas *Microbacterium* sp. and *E. cloacae* exerted the most significant avoidance effects. The analysis of the mVOCs further highlighted the significance of aldehyde compounds, notably 2,3,6-trichlorobenzaldehyde, and alkane compounds, such as eicosane, in mediating the repellency and attraction effects. These results contribute to a deeper understanding of the invasion mechanism of *L. invasa* and provide a scientific basis for developing novel biopesticides or elicitors.

## 1. Introduction

*Leptocybe invasa* Fisher and La Salle (Hymenoptera: Eulophidae) is an invasive pest with a widespread global presence [1]. In recent years, *L. invasa* has posed a significant threat as an emerging invasive pest, severely impacting China’s eucalyptus plants [2]. Robust environmental adaptability, diverse reproductive tactics, diminutive size, gall-induced protection, and overlapping life cycles characterize this insect species. Upon invading a eucalyptus planting area, this pest quickly inflicts damage by consuming all the young leaves and inducing the rapid formation of galls on their twigs and petioles [3,4].

Insects host diverse microorganisms, encompassing bacteria, fungi (including yeasts), and viruses, which have coevolved synergistically over extended periods, forming intricate micro-ecosystems [5]. Under basal circumstances, microorganisms usually establish a dynamic equilibrium among themselves and with the host insects, resulting in a balance that significantly contributes to maintaining the insects’ basal physiological functions [6,7,8]. It is well-known that bacteria pervade the insect realm, encompassing both the cutaneous associates and endosymbiotic bacteria within the organism. These resident microorganisms, dwelling within the insect’s body, can be categorized based on their relationship with the host into probiotics, pathogens, and intermediate flora [9,10,11]. Through long-term co-evolutionary processes, insects have developed intimate relationships with bacteria that facilitate host survival, reproduction, defense against natural enemies, and the elicitation of various physiological responses in the host [5,7,12]. Insect symbiosis has garnered significant attention, emerging as a critical research focus today. Presently, the roles of insect symbiotic bacteria encompass providing nutrients and metabolic support to the host, regulating the host’s environmental adaptation, influencing the invasiveness of the host, bolstering the host insect’s resilience to biotic stresses, modulating the reproduction of the host insect, and augmenting the host’s resistance to pesticides [12]. Considering the substantial ecological and economic implications of *L. invasa* in eucalyptus systems, a better understanding of this species’ biology and behavior becomes imperative. Such research would help to shed light on the mechanisms underlying *L. invasa*’s great invasion capacities and survival strategies and, therefore, inform targeted management interventions to mitigate its devastating effects on eucalyptus plantations.

The detection of olfactory signals is vital for insect survival [13] as they employ sophisticated chemosensory systems to decipher environmental odors, facilitating inter-population communication, establishing predation relationships, and acquiring crucial information on nutrient sources, mating partners, and habitat fitness, thereby rendering volatile compounds central to insect survival strategies [14,15,16]. These signals yield profound insights into the micro-behavioral dynamics within ecosystems [17], and extensive research highlights the pivotal role of microbial Volatile Organic Compounds (mVOCs) in modulating essential insect behaviors such as foraging, mating, and oviposition [18,19,20,21,22]. For example, the mVOCs from *Beauveria bassiana* influenced the oviposition in *Spodoptera frugiperda* (Lepidoptera) [23]. In contrast, the mVOCs from the yeast *Metschnikowia reukaufii* and the bacterium *Asaia astilbes* affected *Bombus impatiens* and its feeding behavior, with the bumblebee showing a preference for the mixture for the habitat and the yeast for nectar [24]. It has also been demonstrated that specific agricultural pest endosymbionts can attract hosts. For instance, *Staphylococcus aureus* has been shown to attract Mexican fruit flies, *Anastrepha ludens* [25]. Culturable bacteria from *Bactrocera dorsalis* (Tephritidae), including *Enterobacteriaceae*, *Enterococcaceae*, and *Bacillaceae*, produce mVOCs that attract more adults of the same species [26]. Endosymbionts like *Enterobacter cloacae* and *Klebsiella pneumoniae* exhibit high attractiveness to *bactrocera zonata* [27], illustrating mVOCs’ role in directing foraging, mate selection, and population patterns [12]. Additionally, numerous hymenopteran wasps utilize mVOCs from their prey or the surrounding environment to locate food sources for their offspring [24]. For instance, the hyperparasitoid enemies of parasitic wasps can reliably use the odors of parasitized caterpillars to find the parasitic wasp larvae developing inside the caterpillar [28]. The mVOCs emitted by certain fungi facilitate host localization over long distances, whereas bacteria and yeasts may contribute to short-distance localization [29].

Previous research has found that, upon invasion by *L. invasa*, eucalyptus trees mount a defense response characterized by producing a substantial array of secondary metabolites to deter the pest. Intriguingly, particular bacteria hosted by *L. invasa* can enzymatically degrade these eucalyptus-derived defensive compounds, effectively assisting the pest in circumventing the host resistance and ultimately achieving pervasive colonization [30]. A scholarly inquiry has predominantly centered around *L. invasa*’s biological attributes, invasion strategies, and host-adaptation mechanisms [31,32,33,34]. The research on the endogenous bacteria and their host *L. invasa*, particularly the influence of cultivable bacterial strains and their emitted mVOCs on the host behavioral responses, remains relatively scarce, with no comprehensive studies documented thus far.

This study focuses on the cultivable endogenous bacteria of *L. invasa* as the subject of investigation [35,36] to identify bacterial strains that significantly influence the behavioral choices of *L. invasa* and characterize the specific mVOCs involved in these processes. We hypothesized that these bacteria, which are widespread in the host, may respond differently to the host even when different bacterial species are closely related and that the species of bacteria may influence the behavioral choices of the host insect by releasing different mVOCs. Ultimately, these mVOCs could shape the behavioral decision-making processes of *L. invasa*. This study employs a Y-tube olfactometer setup to systematically observe and quantitatively assess the behavioral responses of female *L. invasa* insects when exposed to mVOCs, thereby revealing their tendencies to be attracted to or repelled by distinct mVOCs. Simultaneously, gas chromatography–mass spectrometry (GC–MS) technology is utilized to elucidate these mVOCs’ chemical constitution and pinpoint the chemically informative constituents significantly influencing *L. invasa*’s behavioral reaction. Our objective is to comprehensively elucidate the bacterial strains with a significant impact on *L. invasa*, as well as the mVOCs they produce, and subsequently identify mVOCs with the potential for the development of attractants or repellents, thereby providing scientific underpinnings for the sustainable management of *L. invasa*.

## 2. Materials and Methods

### 2.1. Preparation of Experimental Materials

Nine dominant endogenous bacterial strains were isolated and cultured from female specimens of *L. invasa*. The isolated strains included Actinobacteria, Proteobacteria, and Firmicutes [36]. The information about the bacteria is shown in Table 1. All these strains were maintained in the Forest Protection Laboratory within the College of Forestry at Guangxi University (CN). Newly emerged *L. invasa* females were collected from the Teaching and Experimental Base of the College of Forestry at Guangxi University (108°17′ E, 22°51′ N). The host plant for these wasps was identified as the hybrid clone *Eucalyptus grandis × Eucalyptus tereticornis*, precisely the DH201-2 variety, provided by the Guangxi Academy of Forestry Sciences (108.35′ E, 22.92′ N).

### 2.2. Cultivation of Bacteria

The procedure described involves inoculating and cultivating a preserved bacterial strain in LB (Luria-Bertani Medium). LB was utilized, with each liter containing 10 g of tryptone, 5 g of yeast extract, and 10 g of NaCl dissolved in 950 milliliters of deionized water. The pH was adjusted to 7.0 using five mol/L NaOH, and the volume was adjusted to 1 L with deionized water. The prepared LB medium was sterilized in an autoclave (DGL-35G, LICHEN Technology Co., Shaoxing, China) for 20 min. Initially, the strain was used in a sterile inoculation loop inoculated into a centrifuge tube with 5 mL of LB and incubated overnight (dark conditions) for 18 h in a constant temperature shaker set at 28 °C and 220 rpm. Subsequently, a sterile pipette was used to withdraw 0.05 mL of the bacterial suspension and re-inoculate it into a 250 mL conical flask that contained 50 mL of LB. This culture was then incubated in the shaking incubator for 24 h under the same conditions of 28 °C and 220 rpm. Meanwhile, an uninoculated LB was maintained under identical cultivation conditions.

### 2.3. Behavioral Assays of L. invasa

The behavioral responses of *L. invasa* female insects to the endogenous bacteria were evaluated using a transparent glass Y-tube olfactometer. The system consists of two 15 cm long arms forming a 60-degree angle between them and a final stem of 20 cm. The inner diameter of the tubes was 1 cm. Air, propelled by a pump, sequentially passed through a flow meter, a filter, a humidifier, a splitter, and odorant-containing bottles before being distributed into the two arms of the Y-tube. A 1 mL bacterial suspension volume (5 × 10^8^ cells/mL) was pipetted onto sterile cotton wool and placed inside the scent source bottle. Sterile cotton wool soaked with LB served as a control. Once all components were connected, the air pump was activated, and the gas flow rate was set to 30 mL/min using a flow meter. After 5 min of ventilation, the experiment commenced. Recordings started after a 2 min acclimation period against the wind direction, excluding any *L. invasa* that entered the selection arm within the initial 2 min; such insects were reintroduced into the olfactometer. The experiment consisted of ten test groups, including nine combinations of bacteria + air and one combination of LB + air serving as a control. In each group, 20 female *L. invasa* individuals were observed, and the trials were repeated four times. The microbial odor source was replaced with a new one for each set of experiments; i.e., it was replaced four times. To preclude aggregation behavior during each trial, a single *L. invasa* individual was released per experimental run, allowing it to choose by introducing a second insect for subsequent experimentation. Each female insect was subjected to testing only once. If it traversed the selection arm by at least 3 cm and remained for more than 10 s, its choice was recorded; otherwise, it was documented as not having made a choice. After each trial, the test insects were replaced, and the Y-tube, collection bottles, and scent source bottles were cleaned with 75% ethanol, dried, and reused. Additionally, the orientation of the odor source was alternated. Finally, the number of insects choosing different odor sources was recorded. The experiments were performed during two time slots: 8:00–10:00 a.m. and 4:00–7:00 p.m., under constant room temperature and a light intensity of 260 lux.

### 2.4. Collection of mVOCs

VOCs emitted by the nine strains of endogenous bacteria were collected using the Dynamic Headspace Sampling Method [37]. The operations are as follows: first, each bacterial strain was inoculated into 250 mL conical flasks containing 50 mL of LB and incubated at 28 °C constant agitation at 220 rpm for 24 h. This was completed to ensure ample induction of bacterial metabolism to produce mVOCs. Simultaneously, an equivalent volume of uninoculated LB served as a control. A food-grade polyethylene roasting bag (dimensions 430 mm × 550 mm) was tightly wrapped around the conical flask containing the cultivated bacterial strain. A PTFE flexible hose connected the roasting bag to an atmospheric sampling system, with one end inserted into the bag and the other attached to an adsorption tube filled with Tenax ^®®^ TA (Yichen Technology Co., Ltd., Fujian, China) sorbent tubes. The atmospheric sampler was activated to continuously draw air from within the roasting bag at a steady flow rate of 300 mL/min while introducing clean air filtered through activated carbon to maintain stable internal bag pressure. Sampling for each replicate lasted 10 h, with each treatment repeated three times. Upon completion, the adsorption tubes were repeatedly eluted with 2 mL of chromatographically pure n-hexane. The collected eluates were pooled into pre-labeled round-bottom flasks and concentrated using a rotary evaporator. The water bath was set to 65 °C, with the rotor speed adjusted between 30 rpm and 120 rpm throughout the concentration process. The bath temperature and rotor speed were judiciously modulated as needed to prevent overheating or splashing. Once the eluate was reduced to approximately 2 mL, heating was discontinued, and rotation continued to minimize residual vapor. Finally, the concentrated sample was carefully aspirated into a precooled long-gauge syringe and then transferred into a 1 mL brown glass vial, promptly stored at −20 °C in a refrigerator protected from light.

### 2.5. Identification of mVOCs

MVOCs were determined using gas chromatography–mass spectrometry (7890A, Agilent Technologies, Santa Clara, CA, USA). Nine bacterial groups and one LB were determined, ten groups in total, and six biological replicates per species. A 5% Phenyl Polysilphenylene-siloxane (30 m × 0.25 mm × 0.25 μm; Agilent Technologies) column was employed, and 1 µL of the concentrated sample was introduced into the gas chromatograph via non-split injection. The helium of 99.9% purity served as the carrier gas, delivered at a 1.0 mL/min flow rate, while the precolumn pressure was maintained at 7.0699 Psi. The initial column temperature was set to 45 °C for a 10 min hold, followed by a rapid ramp to 100 °C at a rate of 3 °C/min for 1 min, then to 150 °C at a rate of 5 °C/min for 5 min, and ultimately to 250 °C at a rate of 10 °C/min for a final 10 min hold. Under the operating conditions for mass spectrometry, the electron ionization source was employed with an electron energy of 70 eV. The interface temperature was set to 250 °C, the ion source temperature to 230 °C, the quadrupole rod temperature to 150 °C, and the mass scanning range was set to 20~500 *m*/*z* using full scan mode, and mVOCs were automatically searched using the NIST 2014 (National Institute of Standards and Technology, Mass Spectral) library, with tentative identifications of mVOCs achieved by comparing standard mass spectra and retention times. The relative percentage content of each volatile was calculated using peak area normalization [38].

### 2.6. Statistical Analysis

The experimental data were processed and analyzed using SPSS 22.0 (IBM Corporation, Armonk, NY, USA), while Hiplot was utilized for graphing [39]. We compared the total number of females making different choices (bacteria or LB) to the total number of females in each test group, i.e., dividing the number of females making a choice by the 20 females per test and converting it to a percentage. Ultimately, we obtained the mean selection rate by combining data from four replicates and used a *t*-test test to determine whether selection rates differed significantly between treatment groups [40]. Subsequently, after performing a one-way Analysis of Variance (ANOVA) on the response data of the compounds to ascertain if there existed any overall differences among the various compounds, a subsequent Tukey’s Honestly Significant Difference (HSD) multiple comparison test was carried out to discern and statistically distinguish those compounds displaying significant variations among them [41]. We used K-means clustering heatmaps to analyze mVOCs’ profiles (i.e., identify compounds common and specific to bacterial strain).

## 3. Results

### 3.1. L. Response of L. invasa to Different Endogenous Bacteria

**Figure 1:**
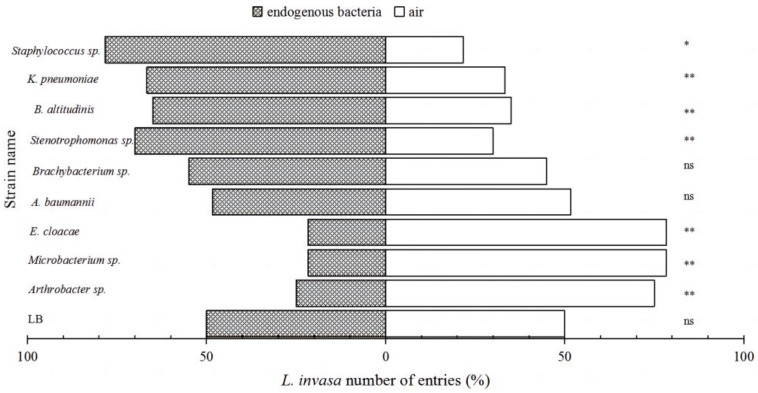
Behavioral selection of female *L. invasa* towards mVOCs from different endogenous bacteria. Note: “**” indicates highly significant differences with *p* < 0.01; “*” indicates with *p* < 0.05; “ns” denotes no significant difference.

Overall, a tiny proportion of insects (less than 0.01%) were found to have made no choice. There were significant differences in the overall response of female *L. invasa* to the nine endogenous bacterial strains, as depicted in Figure 1. Four bacterial strains—*Stenotrophomonas* sp. (*p* = 0.00061), *B. altitudinis* (*p* = 0.00183), *K. pneumoniae* (*p* = 0.00145), and *Staphylococcus* sp. (*p* = 0.01247)—demonstrated a significantly attractive effect on female *L. invasa;* we named them “attractive” strains. Among these, *Staphylococcus* sp. exhibited the highest attraction rate (78.33% of the wasps chose this bacterial strain versus the control), followed by *Stenotrophomonas* sp., *B. altitudinis*, and *K. pneumoniae*, with attraction rates of 70.00%, 65.00%, and 66.67%, respectively. In contrast, the female *L. invasa* exhibited reduced attraction to the mVOCs produced by *Arthrobacter* sp. (*p* = 0.00026), *Microbacterium* sp. (*p* = 0.00002), and *E. cloacae* (*p* = 0.00171) with relative proportions of 25.00%, 21.67%, and 21.67%, and we collectively referred to these three strains as “avoided” strains. For the remaining two bacterial strains, *A. baumannii* (*p* = 0.2302) and *Brachybacterium* sp. (*p* = 0.5342), the overall response proportions of the female *L. invasa* to their respective mVOCs were 48.33% and 45.00%, which did not exhibit a statistically significant difference compared to the LB (*p* = 0.070); these strains were classified as “neutral” strains.

### 3.2. Identification Results of mVOCs

**Figure 2:**
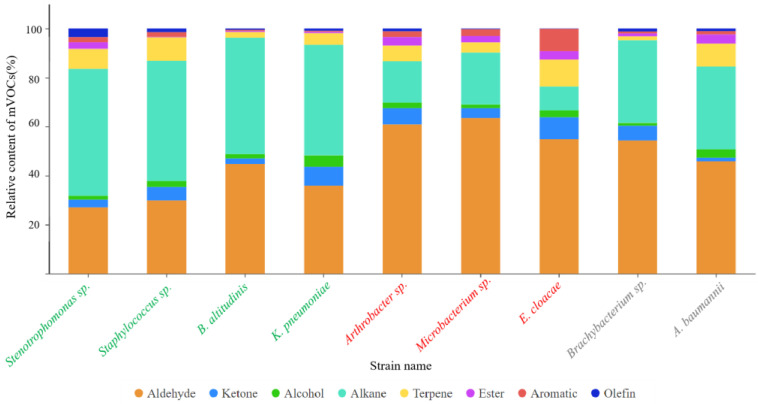
Stacked barplot showing the relative content (%) of the mVOCs emitted by nine endogenous bacterial strains, divided into eight distinct chemical classes. Note: “attractive” strains were identified in green, “avoided” strains were identified in red, and “neutral” strains were identified in gray.

We analyzed the mVOCs produced by nine different bacterial strains, identifying forty-one mVOCs (Table A1), which we categorized into eight distinct chemical classes (Figure 2). Notably, the aldehydes and alkanes had the highest percentage of total abundance among all the compounds, about 46.32% and 34.28%. Following these are terpenes and ketones, whose combined relative content amounted to roughly 6.0% and 5.5%, respectively. As shown in Figure 2, the aldehydes exhibited a significantly higher relative content in the “avoided” strains than other bacterial strains, whereas their content was relatively lower in the “attractive” strains. For the “neutral” strains, the relative content of both the alkanes and aldehydes fell between those of the “attractive” and “avoided” strains.

The “attractive” strains, comprising *Stenotrophomonas* sp., *B. altitudinis*, *K. pneumoniae*, and *Staphylococcus* sp., exhibited alkane contents of 18.9%, 17.8%, 17.3%, and 16.4%, and aldehyde contents of 7.3%, 8.0%, 12.0%, and 9.7% for all the compounds, respectively. In comparison, the “avoided” strains, including *Arthrobacter* sp., *Microbacterium* sp., and *E. cloacae*, showed alkane compositions of 6.1%, 7.7%, and 2.5%, along with aldehyde contents of 16.4%, 17.1%, and 14.8% for all the compounds, respectively. The “neutral” strains, represented by *A. baumannii* and *Brachybacterium* sp., displayed alkane and aldehyde levels at 12.3% (for both strains) and 14.6% and 12.4% for all the compounds, respectively.

Upon the closer examination of the individual strains, it was discovered that *K. pneumoniae* and *E. cloacae* emitted a great amount of ketones, constituting 17.5% and 20.2% of the total ketone content, respectively. Meanwhile, *Staphylococcus* sp. and *E. cloacae* possessed relatively high terpene contents, representing 20.0% and 23.0% of the total terpenes. Intriguingly, despite sharing this trait of high content, these strains exhibited contrasting effects on *L. invasa*: *E. cloacae* demonstrated avoidance properties, whereas *K. pneumoniae* and *Staphylococcus* sp. exhibited attractive effects. Moreover, the “avoided” strain *E. cloacae* contained 45.8% of all the aromatics, while the “attractive” strain *Stenotrophomonas* sp. harbored 38.6% of all the olefins.

### 3.3. Identification of Common mVOCs

**Figure 3:**
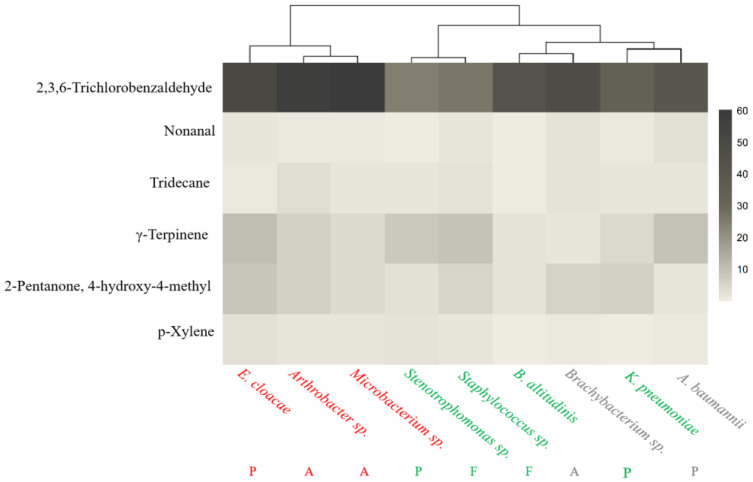
Heat map showing clusters of mVOCs commonly present in nine strains. Note: the horizontal coordinate is the name of the bacteria, and the vertical coordinate is the relative content of the shared mVOCs; the darker the color in the graph, the higher the relative content; A: Actinobacteria, P: Proteobacteria, and F: Firmicutes; “attractive” strains were identified in green, “avoided” strains were identified in red, and “neutral” strains were identified in gray.

We reported the relative content of all the mVOCs within each bacterial strain alongside the statistically significant differences (Table A1). Of particular interest, a conserved set of six compounds were identified across the entire panel of nine bacterial species. These ubiquitous mVOCs were visually represented in their relative content among the nine bacteria and subsequently subjected to K-means clustering (Figure 3). Notably, the resulting clustering configuration aligned well with *L. invasa*’s behavioral preferences inferred from the olfactory cues, with clusters forming around the “avoided” strains in one group, and the “attractive” and “neutral” strains showing similar chemical profiles. Figure 3 showed that two bacterial species from the Firmicutes phylum (*Staphylococcus* sp. and *B. altitudinis*) produced comparable levels of shared mVOCs, both eliciting an attractive effect on the host. Conversely, *L. invasa* exhibited three distinct behavioral responses towards four bacteria classified under Proteobacteria.

Figure 3 illustrated that the concentration of 2,3,6-trichlorobenzaldehyde (an aldehyde compound) was significantly higher in the “avoided” strains compared to the “attractive” ones, while the “neutral” strains exhibited levels intermediate between the two. Nonanal reached its highest concentrations in the neutral strains *Brachybacterium* sp. and *A. baumannii*. γ-terpinene was found in notably high amounts across “attractive” strains such as *Stenotrophomonas* sp. and *Staphylococcus* sp., the “avoided” strain *E. cloacae*, and the neutral strain *A. baumannii*. While 2-hydroxy-4-methyl-2-pentanone predominantly attained its peak levels in the “avoided” strains *E. cloacae* and *Arthrobacter* sp., its presence in the “attractive” strain *K. pneumoniae* was also considerable. In contrast, tridecane and p-xylene exhibited minimal variations in concentration across all the strains, with no statistically significant differences discernible.

### 3.4. Identification of Strain-Specific mVOCs

**Figure 4:**
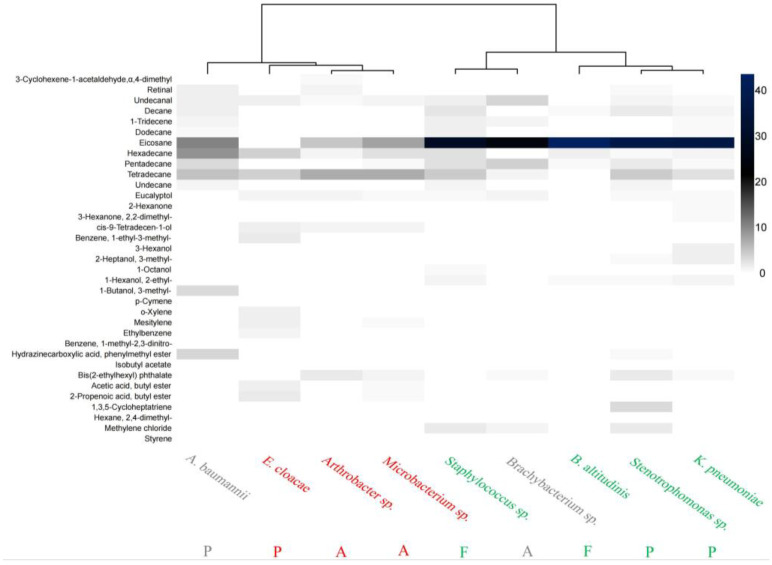
Clustering heat map of mVOCs unique to the nine strains. Note: the horizontal coordinate is the name of the bacteria, and the vertical coordinate is the relative content of the shared mVOCs; the darker the color in the graph, the higher the relative content; the white part of the figure indicates that the strain did not produce this mVOC; A: Actinobacteria, P: Proteobacteria, and F: Firmicutes; “attractive” strains were identified in green, “avoided” strains were identified in red, and “neutral” strains were identified in gray.

In the K-means clustering analysis of the specific mVOCs, we observed the emergence of branch configurations deviating from those derived from the common mVOCs; for instance, the clustering structure of the unique mVOCs produced by Firmicutes changed. Nonetheless, the “avoided” and “attractive” strains consistently clustered into two distinct groups without overlap. Drawing from Table A1 and Figure 4, several key insights emerged. When comparing across all the strains, eicosane exhibited the highest relative content in the “attractive” strains, highlighting its significant role in these organisms. Conversely, tetradecane and ethylbenzene showed peak relative content in the “avoided” strains, underscoring their crucial function in conferring repellency. This evidence underscored the differential importance of these compounds in defining the behavioral responses elicited by the bacterial strains.

Moreover, in conjunction with Table A1, we discovered the existence of three distinctive compounds—cis-9-tetradecen-1-ol, acetic acid butyl ester, and 2-propenoic acid butyl ester—that were exclusive to the “avoided” strains, specifically *Arthrobacter* sp., *Microbacterium* sp., and *E. cloacae*. Conversely, a singular compound, 1-hexanol-2-ethyl, was uniquely found in the “attractive” strains comprising *Stenotrophomonas* sp., *B. altitudinis*, *K. pneumonia, and Staphylococcus* sp.

From the perspective of the individual strains, we particularly noted certain compounds that were unique to those strains and undetected in others despite being present at lower concentrations. For instance, among the strains that exhibited “avoidance” characteristics, the unique mVOCs of *Arthrobacter* sp. comprised 1-methyl-2,3-dinitrobenzene and isobutyl acetate, whereas, for *E. cloacae*, the distinct compound was 1-ethyl-3-methylbenzene. Regarding the “attractive” strains, the presence of 1,3,5-cycloheptatriene marked *Stenotrophomonas* sp. as its unique mVOC; *Staphylococcus* sp. not only possessed 1,3,5-cycloheptatriene but also featured 1-octanol; *B. altitudinis*’s unique mVOCs included 2-hexanone, 2,2-dimethyl-3-hexanone, and p-cymene, and, similarly, the list of unique compounds for *K. pneumoniae* encompassed 2-hexanone and 2,2-dimethyl-3-hexanone.

## 4. Discussion

This study identified four “attractive” strains, three “avoided” strains, and two “neutral” strains that affected *L. invasa* differently through insect olfactory behavioral tests. Our investigation revealed that various endogenous bacterial species within *L. invasa* potentially contributed to modulating the host’s behavioral responses. These data may indicate that the mVOCs from this concentration of “attractive” strains (*Stenotrophomonas* sp., *B. altitudinis*, *K. pneumoniae*, and *Staphylococcus* sp.) and “avoided” strains (*Arthrobacter* sp., *Microbacterium* sp., and *E. cloacae*) may have potential attraction or avoidance effects on female *L. invasa*. Several previous studies unveiled different bacterial species’ attraction or avoidance effects and their metabolites on a broad spectrum of insect behaviors [42]. In the case of *Solenopsis invicta*, it was observed that the *Arthrobacter woluwensis* cultured in its nest soil can attract worker ants. In contrast, several Firmicutes bacteria, including *Bacillus*, *Paenibacillus*, *Brevibacillus*, and other species, typically exhibited an avoidance effect on ants [42]. Further studies have shown that *K. oxytoca* and *Citrobacter freundii* significantly lured female stable flies [43]. Our results revealed that the relationship between bacteria within the same phylum and their produced chemical profiles is a complex issue, and our results merely illustrated that two bacteria within Firmicutes exhibited an attractive effect on *L. invasa* and shared similar chemical characteristics. Nonetheless, this observation might represent a coincidental structure due to the small sample size since other bacteria within the same phylum in our experimental outcomes did not display comparable chemical features. This result was consistent with our earlier conjectures. Accordingly, even within the same bacterial phylum, the behavioral effects of these bacteria on the same or different insect species may vary considerably due to other factors. This underscores the complexity and diversity of bacteria in regulating insect behavior [44].

Via the GC–MS analysis, we revealed that the differences in the content of aldehyde and alkane were closely related to the luring or avoiding behavior of *L. invasa*. This demonstrated the vital role of microbial mVOCs in regulating the behavioral choices of insects. From our findings, it was observed that *L. invasa* showed a preference for strains producing high amounts of alkane compounds and avoided those emitting a high concentration of aldehyde compounds. This likely indicates that high levels of alkanes can strongly lure *L. invasa* through olfactory signals, whereas elevated concentrations of aldehydes can deter *L. invasa*. Moreover, terpenes and ketones appeared to potentially cooperate with aldehydes and alkanes, jointly impacting the behavioral preferences of *L. invasa*. Growing evidence suggests that volatile compounds from bacteria can also induce changes in insect behavior through olfactory cues [45]. For instance, the esters, organic acids, aromatic compounds, and cycloalkanes produced by *Corynebacterium sputi* repelled the parasitic behavior of *Aphidius colemani* [44]. Phenylethyl alcohol, another compound emitted by *Staphylococcus xylosus*, is a feeding attractant for *Aphis fabae* [46,47]. However, the content of a single compound class does not entirely determine the behavioral response of insects, and the synergistic effect of different compounds at different concentrations may be the key to deciding the attraction or avoidance of insects.

Although the two K-means clustering analyses of mVOCs yielded different groupings, a consistent observation was the clear distinction between the “avoidance” and “attraction” strains without any crossover, highlighting fundamental disparities between these strain categories regarding the compounds produced and their concentrations. This pattern strongly suggested that the preference of *L. invasa* for a particular strain was jointly governed by the variety and relative content of the compounds secreted by that strain. In this study, we showed that the experimental evidence conclusively identified 2,3,6-trichlorobenzaldehyde as the central mVOC triggering the avoidance response in *L. invasa*. In contrast, nonanal did not appear to exert a significant effect on the behavioral inclination of *L. invasa*. As for γ-terpinene and 2-hydroxy-4-methyl-2-pentanone, their impact on the insect behavior proved more nuanced and multifaceted, implying that these compounds may have played complex regulatory roles in a myriad of interactions between the insect and its environment [48,49]. Notably, 2,3,6-trichlorobenzaldehyde, classified as an aromatic aldehyde, has been documented to stimulate pronounced electroantennogram responses in *Helicoverpa armigera* adults [50]. Correspondingly, an agarose preparation enriched with a wide array of aromatic compounds but devoid of phenolic materials has been found to exhibit trapping effects on *H. armigera* adults [51]. Such a phenomenon may be attributed to the proportion of specific compounds in their mVOCs.

The alkane compounds had a high relative content in the “attractive” strains, suggesting that alkane compounds may serve as crucial signaling molecules eliciting the attraction response of *L. invasa*. Among these, eicosane warranted particular attention. Our study also demonstrated that tetradecane and ethylbenzene likely had a significant avoidance effect on *L. invasa*. Specifically, cis-9-tetradecen-1-ol, butyl acetate, and butyl 2-propenoate were found solely in strains exhibiting avoidance properties, whereas 2-ethyl-1-hexanol was identified uniquely in those attracting the insect. This distribution reinforced the direct correlation between specific compounds and the strains’ tendencies to attract or repel. Moreover, special attention should be paid to certain compounds, such as 1-ethyl-3-methylbenzene, 1,3,5-cycloheptatriene, 2-hexanone, and 2,2-dimethyl-3-hexanone, which, even at low concentrations, maintained strain specificity and potentially enhanced the strains’ appeal to insects. However, the same compounds might have different effects on different species of insects. In the context of host-finding behavior, eicosane extracted from the integument of *Mythimna separata* was found to be instrumental in facilitating the host localization process by the parasitoid *Microplitis mediator* [52]. Similarly, within the suite of cuticular mVOCs obtained from the rice moth, eicosane emerged as a standout compound, demonstrating a significantly heightened attraction potential for its corresponding parasitoid, *Habrobracon hebetor*, surpassing the efficacy of the other constituents examined [53]. In terms of insect behavioral tests, various concentration gradients of eicosanes were evaluated for their avoidance effects on the foragers of *Apis florea*, where a concentration of 10.0% eicosanes attracted *Apis andreniformis* foragers, while 5.0% eicosanes repelled the insects [54]. In subsequent investigations, it was imperative to account for the effects of the culture medium type and pH conditions on the qualitative and quantitative synthesis of mVOCs [55], particularly in light of the prior findings indicating that the generation of mVOCs by common Enterobacteriaceae strains inhabiting the gut of *Bactrocera tryoni* was subject to considerable strain-specific and media-dependent variations, as well as showing semi-quantitative fluctuations depending on the pH levels and duration of incubation [39]. Hence, differences exist in the perceived sensitivity of insects to various compounds, and alterations in the compound concentrations and pH could significantly influence their effects [39,56,57].

An intriguing question arose: “Why would endogenous bacteria exert detrimental effects on host behavior?” We posit that symbiotic bacteria, ubiquitous across diverse insect species, primarily augment the host’s defense against pathogens and parasites [58]. Prior research had established that the endosymbiotic bacteria inhabiting pea aphids (*Acyrthosiphon pisum*) were efficacious in suppressing the growth of parasitic wasp (*Aphidius ervi*) larvae, thereby dramatically enhancing aphid survival following wasp attacks [59]. Furthermore, these endosymbionts contribute to synthesizing vital nutrients, facilitating their hosts’ growth and reproductive success [60]. Nevertheless, the exact functional implications of endosymbionts within *L. invasa* necessitate further exhaustive scientific exploration to unravel.

In summary, this study, utilizing insect behavioral assays coupled with GC–MS analysis, elucidated the intricate relationship between nine bacterial mVOCs and the olfactory-driven behavioral choices of *L. invasa*, thereby improving our comprehension of the role of mVOCs in the ecological modulation of insect behavior. Furthermore, we identified the critical mVOCs with attracting properties: eicosane and 1-hexanol-2-ethyl, as well as those with avoidance effects: 2,3,6-trichlorobenzaldehyde, tetradecane, ethylbenzene, γ-terpinene, cis-9-tetradecen-1-ol, acetic acid butyl ester, and 2-propenoic acid butyl ester. These findings provided a foundational dataset for developing novel eco-friendly attractants or deterrents targeting *L. invasa* and furnished a substantial underpinning for further inquiries into the functional significance of microbial mVOCs in the ecological regulation of insect behavior. While this investigation has established linkages between specific mVOCs and *L. invasa*’s behavioral preferences, a more profound examination is warranted concerning the interplay among compounds, concentration-dependent effects, and environmental variables’ influence on the volatile emissions and insect behavioral responsiveness. Future research endeavors should concentrate on elucidating the dose-dependent effects, synergistic interactions between compounds, ecological factors, as well as conducting practical demonstration studies. Additionally, employing GC-EAG/olfactometry techniques with synthetic standards can facilitate the precise manipulation of the behavior of *Leptocybe invasa*, thereby enhancing our understanding and control strategies.

## Figures and Tables

**Table 1:** Basic information on the nine endogenous bacteria isolated from *L. invasa*.

Phylum	Genus	Strain Name	Gram’s Dyeing
Actinobacteria	*Arthrobacter*	*Arthrobacter* sp.	G+
	*Microbacterium*	*Microbacterium* sp.	G+
	*Brachybacterium*	*Brachybacterium* sp.	G+
Proteobacteria	*Pseudomonas*	*Stenotrophomonas* sp.	G−
	*Klebsiella*	*K. pneumoniae*	G−
	*Acinetobacter*	*A. baumannii*	G−
	*Enterobacter*	*E. cloacae*	G−
Firmicutes	*Staphylococcus*	*Staphylococcus* sp.	G+
	*Bacillus*	*B. altitudinis*	G+

## Data Availability

The data presented in this study are available on request from the corresponding author.

## Appendix A

**Table A1:** Relative content and statistical significance results of 41 mVOCs, illustrated with letter coding; note: data followed by different letters indicate significant differences.

Class	Compound/Bacterial Name	*Arthrobacter* sp.	*Microbacterium* sp.	*E. cloacae*	*A. baumannii*	*Brachybacterium* sp.	*Stenotrophomonas* sp.	*B. altitudinis*	*K. pneumoniae*	*Staphylococcus* sp.
Aldehyde	2,3,6-Trichlorobenzaldehyde	58.11 ± 9.37 ^a^	60.57 ± 2.01 ^a^	51.60 ± 3.75 ^b^	40.42 ± 5.91 ^c^	48.30 ± 9.99 ^bc^	24.87 ± 2.68 ^d^	43.91 ± 8.28 ^c^	33.65 ± 9.93 ^d^	26.39 ± 3.56 ^d^
	3-Cyclohexene-1-acetaldehyde,α,4-dimethyl	0.63 ± 0.15 ^a^	0.27 ± 0.05 ^b^	/	/	/	/	0.08 ± 0.02 _c_	0.06 ± 0.02 ^c^	/
	Nonanal	1.32 ± 0.37 ^b^	1.36 ± 0.32 ^b^	1.66 ± 0.20 ^b^	2.81 ± 0.76 ^a^	2.42 ± 0.19 ^a^	0.53 ± 0.23 ^c^	0.60 ± 0.25 ^c^	1.40 ± 0.30 ^b^	1.84 ± 0.24 ^b^
	Retinal	0.91 ± 0.45 ^b^	0.28 ± 0.13 ^c^	0.26 ± 0.07 ^c^	1.32 ± 0.35 ^a^	/	0.78 ± 0.09 ^b^	/	0.32 ± 0.13 ^c^	0.21 ± 0.06 ^c^
	Undecanal	0.69 ± 0.21 ^c^	1.22 ± 0.21 ^b^	1.33 ± 0.11 ^b^	1.33 ± 0.67 ^b^	3.55 ± 1.98 ^a^	0.93 ± 0.16 ^b^	0.16 ± 0.04 ^d^	0.53 ± 0.16 ^c^	1.41 ± 0.15 ^b^
Alkane	Decane	/	0.31 ± 0.09 ^c^	/	1.40 ± 0.31 ^b^	/	2.14 ± 0.57 ^a^	0.64 ± 0.18 ^bc^	0.93 ± 0.17 ^b^	2.56 ± 0.31 ^a^
	1-Tridecene	0.43 ± 0.13 ^c^	/	/	1.00 ± 0.26 ^b^	1.17 ± 0.58 ^b^	0.17 ± 0.06 ^d^	0.13 ± 0.02 ^d^	0.48 ± 0.06 ^c^	1.43 ± 0.19 ^a^
	Dodecane	/	/	/	0.56 ± 0.26 ^bc^	/	/	0.31 ± 0.11 ^c^	0.79 ± 0.23 ^b^	1.33 ± 0.40 ^a^
	Eicosane	5.06 ± 2.19 ^e^	8.11 ± 2.25 ^de^	/	10.89 ± 2.43 ^d^	25.16 ± 7.24 ^c^	37.92 ± 6.45 ^b^	43.74 ± 11.10 ^a^	37.21 ± 4.51 ^b^	29.50 ± 3.31 ^c^
	Hexadecane	1.02 ± 0.51 ^cd^	2.82 ± 1.34 ^c^	4.17 ± 1.62 ^b^	9.31 ± 2.35 ^a^	/	0.50 ± 0.10 ^d^	1.57 ± 0.31 ^cd^	0.95 ± 0.25 ^d^	2.75 ± 0.59 ^c^
	Pentadecane	/	0.64 ± 0.32 ^d^	/	3.15 ± 0.87 ^b^	4.32 ± 0.73 ^a^	1.86 ± 0.65 ^c^	0.51 ± 0.25 ^d^	0.54 ± 0.20 ^d^	2.68 ± 0.93 ^bc^
	Tetradecane	7.37 ± 2.14 ^a^	7.09 ± 3.08 ^a^	4.19 ± 0.06 ^b^	5.55 ± 1.82 ^b^	0.91 ± 0.43 ^d^	4.71 ± 1.58 ^b^	/	2.84 ± 1.39 ^c^	4.59 ± 0.50 ^b^
	Tridecane	3.37 ± 1.81 ^a^	1.77 ± 0.33 ^b^	1.38 ± 0.88 ^b^	1.81 ± 0.61 ^b^	2.22 ± 0.84 ^b^	1.85 ± 0.51 ^b^	0.65 ± 0.16 ^c^	1.85 ± 0.43 ^b^	2.43 ± 0.51 ^b^
	Undecane	/	/	/	1.07 ± 0.25 ^a^	/	0.93 ± 0.18 ^a^	/	/	1.10 ± 0.35 ^a^
Terpene	Eucalyptol	0.95 ± 0.32 ^ab^	0.72 ± 0.20 ^b^	1.11 ± 0.33 ^a^	/	1.20 ± 0.31 ^a^	0.57 ± 0.14 ^b^	0.43 ± 0.22 ^c^	0.74 ± 0.15 ^b^	0.76 ± 0.22 ^b^
	γ-Terpinene	6.49 ± 1.19 ^ab^	4.20 ± 0.55 ^b^	10.97 ± 3.55 ^a^	9.37 ± 3.03 ^a^	1.58 ± 0.51 ^c^	8.13 ± 1.06 ^a^	2.41 ± 1.05 ^c^	4.68 ± 1.17 ^b^	9.61 ± 1.96 ^a^
Ketone	2-Hexanone	/	/	/	/	/	/	0.13 ± 0.03 ^b^	0.77 ± 0.24 ^a^	/
	2-Pentanone, 4-hydroxy-4-methyl-	6.55 ± 1.53 ^b^	4.04 ± 1.01 ^c^	8.92 ± 0.85 ^a^	1.45 ± 0.67 ^d^	6.07 ± 1.31 ^b^	3.11 ± 1.00 ^c^	2.05 ± 1.58 ^d^	6.49 ± 1.18 ^b^	5.49 ± 1.15 ^b^
	3-Hexanone, 2,2-dimethyl-	/	/	/	/	/	/	0.13 ± 0.03 ^b^	0.48 ± 0.17 ^a^	/
Alcohol	cis-9-Tetradecen-1-ol	1.03 ± 0.13 ^b^	0.88 ± 0.08 ^b^	1.46 ± 0.64 ^a^	/	/	/	/	/	/
	Benzene, 1-ethyl-3-methyl-	/	/	2.18 ± 0.41 ^a^	/	/	/	/	/	/
	3-Hexanol	/	/	/	/	/	/	0.28 ± 0.05 ^b^	1.42 ± 0.23 ^a^	/
	2-Heptanol, 3-methyl-	0.32 ± 0.03 ^bc^	/	/	/	/	0.45 ± 0.06 ^b^	0.40 ± 0.04 ^b^	1.54 ± 0.35 ^a^	0.24 ± 0.02 ^c^
	1-Octanol	/	/	/	/	/	/	/	/	0.46 ± 0.51 ^a^
	1-Hexanol, 2-ethyl-	/	/	/	/	/	0.52 ± 0.15 ^b^	0.66 ± 0.18 ^b^	1.00 ± 0.24 ^a^	1.04 ± 0.25 ^a^
Aromatic	1-Butanol, 3-methyl-	/	/	0.33 ± 0.03 ^b^	3.37 ± 0.15 ^a^	/	/	/	/	/
	p-Xylene	1.72 ± 0.53 ^bc^	1.64 ± 0.16 ^bc^	3.06 ± 0.82 ^a^	1.42 ± 0.71 ^c^	0.85 ± 0.21 ^d^	2.14 ± 0.51 ^b^	0.23 ± 0.07 ^e^	0.43 ± 0.07 ^e^	1.70 ± 0.60 ^bc^
	p-Cymene	/	/	/	/	/	/	0.06 ± 0.02 ^a^	/	/
	o-Xylene	/	/	1.49 ± 0.52 ^a^	/	/	/	/	/	/
	Mesitylene	/	0.81 ± 0.20 ^b^	1.39 ± 0.45 ^a^	/	/	/	/	/	/
	Ethylbenzene	0.39 ± 0.13 ^b^	0.26 ± 0.23 ^b^	0.94 ± 0.23 ^a^	/	/	/	/	/	0.20 ± 0.10 ^b^
	Benzene, 1-methyl-2,3-dinitro-	0.27 ± 0.09 ^a^	/	/	/	/	/	/	/	/
Ester	Hydrazinecarboxylic acid, phenylmethyl ester	0.28 ± 0.14 ^bc^	0.30 ± 0.03 ^bc^	/	3.78 ± 0.12 ^a^	0.33 ± 0.17 ^bc^	0.64 ± 0.16 ^b^	0.08 ± 0.04 ^c^	0.10 ± 0.04 ^c^	0.23 ± 0.08 ^bc^
	Isobutyl acetate	0.23 ± 0.11 ^a^	/	/	/	/	/	/	/	/
	Bis(2-ethylhexyl) phthalate	2.16 ± 0.88 ^a^	1.14 ± 0.32 ^b^	/	/	0.77 ± 0.30 ^c^	2.03 ± 0.05 ^a^	0.40 ± 0.11 ^c^	0.51 ± 0.11 ^c^	/
	Acetic acid, butyl ester	0.43 ± 0.13 ^c^	0.78 ± 0.16 ^b^	1.43 ± 0.19 ^a^	/	/	/	/	/	/
	2-Propenoic acid, butyl ester	0.35 ± 0.05 ^b^	0.46 ± 0.17 ^b^	1.99 ± 0.38 ^a^	/	/	/	/	/	/
Olefin	1,3,5-Cycloheptatriene	/	/	/	/	/	3.30 ± 0.36 ^a^	/	/	0.33 ± 0.03 ^b^
	Hexane, 2,4-dimethyl-	/	0.32 ± 0.07 ^a^	/	/	/	/	0.04 ± 0.01 ^b^	/	0.26 ± 0.09 ^a^
	Methylene chloride	/	/	/	/	1.15 ± 0.59 ^b^	1.92 ± 0.65 ^a^	/	/	1.79 ± 0.40 ^a^
	Styrene	/	/	0.14 ± 0.04 ^b^	/	/	/	0.38 ± 0.09 ^a^	0.30 ± 0.10 ^a^	/

## References

[1] Pereira J.M., De Melo A., Rodrigues O.D., Dias T., Wilcken C.F. (2014). Record of the *Leptocybe invasa* in the state goias, brazil. Cienc. Rural.

[2] Fernandes B.V., Barcelos J., Andrade H.B., Zanuncio J.C. (2014). *Leptocybe invasa* (hymenoptera: eulophidae), an exotic pest of eucalyptus, in minas gerais state, brazil. Fla. Entomol..

[3] Barbosa L.R., Rodrigues A.P., De Souza A.R., Puretz B.D., Wilcken C.F., Zanuncio J.C. (2018). *Leptocybe invasa* (Hymenoptera: eulophidae) on *eucalyptus* seedlings in Santa catarina state, brazil. Cienc. Florest..

[4] Chen H., Yao J., Xu Z. (2009). First description of the male of leptocybe invasa fisher & la salle (hymenoptera: eulophidae) from China. J. Environ. Entomol..

[5] Moya A., Pereto J., Gil R., Latorre A. (2008). Learning how to live together: Genomic insights into prokaryote-animal symbioses. Nat. Rev. Genet..

[6] Genta F.A., Dillon R.J., Terra W.R., Ferreira C. (2006). Potential role for gut microbiota in cell wall digestion and glucoside detoxification in *Tenebrio molitor* larvae. J. Insect Physiol..

[7] Frago E., Dicke M., Godfray H.C.J. (2012). Insect symbionts as hidden players in insect-plant interactions. Trends Ecol. Evol..

[8] Giron D., Dedeine F., Dubreuil G., Huguet E., Mouton L., Outreman Y., Vavre F., Simon J.C., Sauvion N., Thiery D., Calatayud P.A. (2017). Influence of microbial symbionts on plant-insect interactions. Advances in Botanical Research.

[9] Canovas Martinez A.F., De Almeida L.G., Beraldo Moraes L.A., Consoli F.L. (2019). Microbial diversity and chemical multiplicity of culturable, taxonomically similar bacterial symbionts of the leaf-cutting ant acromyrmex coronatus. Microb. Ecol..

[10] Jing X., Wong A.C., Chaston J.M., Colvin J., Mckenzie C.L., Douglas A.E. (2014). The bacterial communities in plant phloem-sap-feeding insects. Mol. Ecol..

[11] Schmid R.B., Lehman R.M., Broezel V.S., Lundgren J.G. (2015). Gut bacterial symbiont diversity within beneficial insects linked to reductions in local biodiversity. Ann. Entomol. Soc. Am..

[12] Chen S., Zhou A., Xu Y. (2023). Symbiotic bacteria regulating insect-insect/fungus/virus mutualism. Insects.

[13] De Bruyne M., Baker T.C. (2008). Odor detection in insects: Volatile codes. J. Chem. Ecol..

[14] Liu W., Liu Y., Wang G. (2018). Advances in the insect olfactory plasticity. J. Environ. Entomol..

[15] Mbaluto C.M., Ayelo P.M., Duffy A.G., Erdei A.L., Tallon A.K., Xia S., Caballero-Vidal G., Spitaler U., Szelenyi M.O., Duarte G.A. (2020). Insect chemical ecology: Chemically mediated interactions and novel applications in agriculture. Arthropod-Plant Interact..

[16] Thomas G., Rusman Q., Morrison W.R., Magalhaes D.M., Dowell J.A., Ngumbi E., Osei-Owusu J., Kansman J., Gaffke A., Damodaram K. (2023). Deciphering plant-insect-microorganism signals for sustainable crop production. Biomolecules.

[17] Ren B., Luo W., Zhang X., Wang Y. (2017). A review of the research on insect olfactory communications. J. Jilin Agric. Univ..

[18] Isidorov V., Zalewski A., Zambrowski G., Swiecicka I. (2023). Chemical composition and antimicrobial properties of honey bee venom. Molecules.

[19] Plaza J.J.G., Hradecky J. (2023). The tropical cookbook: Termite Diet and phylogenetics-over Geographical origin-drive the Microbiome and functional genetic structure of Nests. Front. Microbiol..

[20] Chouati T., Maski S., Melloul M., Ajdig M., Ouchari L., Rached B., El Fahime E. (2023). Draft genome sequence of a mosquito repellent *Bacillus licheniformis* strain ba1 isolated from desert soil. Microbiol. Resour. Ann..

[21] Noel A., Dumas C., Rottier E., Beslay D., Costagliola G., Ginies C., Nicole F., Rau A., Le Conte Y., Mondet F. (2023). Detailed chemical analysis of honey bee (*Apis mellifera*) worker brood volatile profile from egg to emergence. PLoS ONE.

[22] Meiners T. (2015). Chemical ecology and evolution of plant-insect interactions: A multitrophic perspective. Curr. Opin. Insect Sci..

[23] Ramirez-Ordorica A., Contreras-Cornejo H.A., Orduno-Cruz N., Luna-Cruz A., Winkler R., Macias-Rodriguez L. (2022). Volatiles released by beauveria bassiana induce oviposition behavior in the fall armyworm spodoptera frugiperda (lepidoptera: Noctuidae). Fems Microbiol. Ecol..

[24] Schaeffer R.N., Rering C.C., Maalouf I., Beck J.J., Vannette R.L. (2019). Microbial Metabolites Elicit Distinct Olfactory And Gustatory Preferences In Bumblebees. Biol. Lett..

[25] Robacker D.C., Flath R.A. (1995). Attractants fromstaphylococcus aureus cultures for mexican fruit fly, anastrepha ludens. J. Chem. Ecol..

[26] Wang H., Jin L., Peng T., Zhang H., Chen Q., Hua Y. (2014). Identification of cultivable bacteria in the intestinal tract of *Bactrocera dorsalis* from three different populations and determination of their attractive potential. Pest. Manag. Sci..

[27] Reddy K., Sharma K., Singh S. (2014). Attractancy potential of culturable bacteria from the gut of peach fruit fly, *Bactrocera zonata* (saunders). Phytoparasitica.

[28] Bourne M.E., Gloder G., Weldegergis B.T., Slingerland M., Ceribelli A., Crauwels S., Lievens B., Jacquemyn H., Dicke M., Poelman E.H. (2023). Parasitism causes changes in caterpillar odours and associated bacterial communities with consequences for host-location by a hyperparasitoid. PLoS Pathog..

[29] Davis T.S., Crippen T.L., Hofstetter R.W., Tomberlin J.K. (2013). Microbial volatile emissions as insect semiochemicals. J. Chem. Ecol..

[30] Kou J., Liu F., Liu Y., Ma L., Lu M. (2020). The associated bacteria of leptocybe invasa fisher & la salle (hymenoptera: eulophidae) facilitate their host to overcome eucalyptus chemical defense. J. Biosaf..

[31] Zhang H., Song J.Y., Zhao H.X., Li M., Han W.H. (2021). Predicting the distribution of the invasive species *Leptocybe invasa*: Combining maxent and geodetector models. Insects.

[32] Eskiviski E.R., Schapovaloff M.E., Dummel D.M., Fernandez M.M., Aguirre F.L. (2018). Susceptibility of eucalyptus species and hybrids to the gall wasp *Leptocybe invasa* (hymenoptera: eulophidae) in northern misiones, argentina. For. Syst..

[33] Nunes T.V., Rodrigues J.N., Pinto I.O., Pimenta R.S., Sarmento M.I., Silva R.S., Souza P., De Souza D.J., Joseph L.A., Souza M. (2023). Endophytic development of the entomopathogenic fungus *Beauveria bassiana* reduced the development of galls and adult emergence of *Leptocybe invasa* in susceptible *eucalyptus*. Sustainability.

[34] ZHU F., HAN P., REN S., PENG Z., WAN F. (2011). Effect of host plants on adult biology of leptocybe invasa. Chin. J. Appl. Entomol..

[35] Guo C., Peng X., Zheng X., Wang X., Wang R., Huang Z., Yang Z. (2020). Comparison of bacterial diversity and abundance between sexes of *Leptocybe invasa* fisher & la salle (hymenoptera: eulophidae) from China. PeerJ.

[36] Guo C.H., Peng X., Wang H.T., Zheng X.L., Hu P., Zhou J., Ding Z.R., Wang X., Yang Z.D. (2021). Bacterial diversity of *Leptocybe invasa* fisher & la salle (hymenoptera: eulophidae) from different geographical conditions in China. Arch. Insect Biochem..

[37] Psoma A., Anastasaki E., Partsinevelos G., Milonas P. (2023). Isolation and identification of volatile compounds from a protein-based food lure: Electrophysiological and behavioral responses of *Bactrocera oleae* adults. Chemoecology.

[38] Tallon A.K., Manning L.A., Mas F. (2023). Electrophysiological and behavioral responses of virgin female *Bactrocera tryoni* to microbial volatiles from enterobacteriaceae. Microorganisms.

[39] Li J., Miao B., Wang S., Dong W., Xu H., Si C., Wang W., Duan S., Lou J., Bao Z. (2022). Hiplot: A comprehensive and easy-to-use web service for boosting publication-ready biomedical data visualization. Brief. Bioinform..

[40] Arechavaleta-Velasco M.E., Hunt G.J. (2004). Binary trait loci that influence honey bee (hymenoptera: apidae) guarding behavior. Ann. Entomol. Soc. Am..

[41] Dong Z., Ge F. (2013). Statistical analysis of insect population data and the use of spss. Chin. J. Appl. Entomol..

[42] Travanty N.V., Vargo E.L., Schal C., Apperson C.S., Ponnusamy L. (2022). Bacterial isolates derived from nest soil affect the attraction and digging behavior of workers of the red imported fire ant, *Solenopsis invicta* buren. Insects.

[43] Hadapad A.B., Prabhakar C.S., Chandekar S.C., Tripathi J., Hire R.S. (2016). Diversity of bacterial communities in the midgut of *Bactrocera cucurbitae* (diptera: tephritidae) populations and their potential use as attractants. Pest. Manag. Sci..

[44] Goelen T., Sobhy I.S., Vanderaa C., De Boer J.G., Delvigne F., Francis F., Wäckers F., Rediers H., Verstrepen K.J., Wenseleers T. (2020). Volatiles of bacteria associated with parasitoid habitats elicit distinct olfactory responses in an aphid parasitoid and its hyperparasitoid. Funct. Ecol..

[45] Ameye M., Allmann S., Verwaeren J., Smagghe G., Haesaert G., Schuurink R.C., Audenaert K. (2018). Green leaf volatile production by plants: A meta-analysis. New Phytol..

[46] Ivens A.B.F., Gadau A., Kiers E.T., Kronauer D.J.C. (2018). Can social partnerships influence the microbiome? insights from ant farmers and their trophobiont mutualists. Mol. Ecol..

[47] Fischer C.Y., Lognay G.C., Detrain C., Heil M., Grigorescu A., Sabri A., Thonart P., Haubruge E., Verheggen F.J. (2015). Bacteria may enhance species association in an ant-aphid mutualistic relationship. Chemoecology.

[48] Hou Z., Wang X., Wu H., An Y., Zhang J., Wang J., Liang J. (2022). Control Effects of Elsholtzia fruticose Essential Oil and Its Main Component gamma-terpinene Against the Adults and Larvae of Tribolium castaneum. J. Chin. Cereals Oils Assoc..

[49] Diksh, Singh S., Mahajan E., Sohal S.K. (2023). Growth inhibitory, immunosuppressive, cytotoxic, and genotoxic effects of γ-terpinene on Zeugodacus cucurbitae (Coquillett) (Diptera: Tephritidae). Sci. Rep..

[50] Yan Z., Yan Y., Kang L., Wang C. (2006). EAG responses of campoletis chlorideae uchida to plant volatiles and host pheromone gland compounds. Acta Entomol. Sin..

[51] Li W.Z., Yuan G.H., Sheng C.F., Guo X.R. (2005). Active compounds in *Populus nigra* L. wilted leaves responsible for attracting *Helicoverpa armigera* (hubner) (lep., noctuidae) and new agaropectin formulation. J. Appl. Entomol..

[52] Tao Y., Shan S., Wang S., Li R., Zhang Y. (2023). Volatile components from the body surface of oriental armyworm mythimna separata larvae and their effects on the host location behavior of parasitoid wasp microplitis mediator. Acta Phytophylacica Sin..

[53] Gowda G.B., Adak T., Jayanthi P., Kumar P.S., Guru-Pirasanna-Pandi G., Patil N.B., Annamalai M., Rath P.C. (2023). Volatile cues from *Corcyra cephalonica* larva elicit behavioural responses in parasitoid, *Habrobracon hebetor*. Curr. Sci. India.

[54] Suwannapong G., Benbow M.E., Chinokul C., Seanbualuang P., Sivaram V. (2011). Bioassay of the mandibular gland pheromones of *Apis florea* on the foraging activity of dwarf honey bees. J. Apic. Res..

[55] Hasnain M., Saeed S., Ullah U.N., Ullah S., Zaka S.M. (2023). Synergist response of the peach fruit fly, bactrocera zonata (saunders) to some ammonium based proteinaceous food bait attractants. BMC Zool..

[56] Younas A., Waris M.I., Ul Qamar M.T., Shaaban M., Prager S.M., Wang M. (2018). Functional analysis of the chemosensory protein msepcsp8 from the oriental armyworm *Mythimna separata*. Front. Physiol..

[57] Cannon W.N.J. (1990). Olfactory response of eastern spruce budworm larvae to red spruce needles exposed to acid rain and elevated levels of ozone. J. Chem. Ecol..

[58] Eleftherianos I., Atri J., Accetta J., Castillo J.C. (2013). Endosymbiotic bacteria in insects: Guardians of the immune system?. Front. Physiol..

[59] Frago E., Mala M., Weldegergis B.T., Yang C., McLean A., Godfray H.C.J., Gols R., Dicke M. (2017). Symbionts protect aphids from parasitic wasps by attenuating herbivore-induced plant volatiles. Nat. Commun..

[60] Koga R., Tsuchida T., Fukatsu T. (2003). Changing partners in an obligate symbiosis:: A facultative endosymbiont can compensate for loss of the essential endosymbiont *Buchnera* in an aphid. Proc. R. Soc. B-Biol. Sci..

